# Residence with a Person Who Used Substances and Childhood Anxiety and Depression: A Cross-Sectional Analysis of the 2019 National Health Interview Survey

**DOI:** 10.3390/children9091296

**Published:** 2022-08-26

**Authors:** Zarena Jafry, Kenneth Chui, Thomas J. Stopka, Laura Corlin

**Affiliations:** 1 Department of Public Health and Community Medicine, Tufts University School of Medicine, Boston, MA 02111, USA; 2 Tufts Clinical Translational Science Institute, Boston, MA 02111, USA; 3 Department of Civil and Environmental Engineering, Tufts University School of Engineering, Medford, MA 02155, USA

**Keywords:** anxiety, depression, children, adolescents, substance use

## Abstract

Background: Children who live with a parent with a substance use disorder (SUD) are more likely to experience adverse health outcomes, including mental health disorders. We assessed whether residing with anyone who used substances was associated with children’s anxiety and/or depression, and whether these associations differed by the children’s age or sex. Methods: We analyzed nationally representative cross-sectional data from the 2019 National Health Interview Survey (n = 6642). The associations between ever residing with someone who used substances and caregiver-reported children’s anxiety and depression frequency (never/a few times a year/monthly/weekly/daily) were estimated using multinomial logistic regression models, adjusted for children’s age, children’s sex, children’s race/ethnicity, annual household income, and highest educational attainment by an adult in the household. We assessed whether the associations differed based on the children’s age (5–11/12–17 years) or sex. Results: Children who had resided with someone who used substances were more likely to be reported by their caregiver as having daily anxiety (risk ratio (RR) = 2.84; 95% confidence interval (CI) = 2.04, 3.95; referent = never anxious) and daily depression (RR = 3.35; 95% CI = 1.98, 5.67; referent = never depressed). Associations with more frequent anxiety were stronger among adolescents than younger children. Associations between residing with someone who used substances and depression frequency differed based on children’s age and sex. Conclusions: Our results suggest that residing with someone who used substances is associated with children’s anxiety and depression. Our findings can help inform screening and treatment efforts for anxiety and depression among children, as well as for the person using substances.

## 1. Introduction

A substance use disorder (SUD) is characterized by the inability to control one’s use of legal or illegal drugs or medications, including, but not limited to, alcohol, nicotine, and pain control medications [1]. In the United States (U.S.), 12.3% of children aged 17 or younger (8.7 million children) live with at least one parent with a SUD [1], and it is unknown how many children live with other household members with a SUD. These children are more likely to develop a SUD themselves [2,3,4,5,6], and to live in traumatic environments [3], experience social difficulties [1,7,8], parental abuse [1,9,10,11,12], and stress [13,14]. Children with at least one parent with a SUD are also more likely to develop mental health and/or behavioral disorders [9,15,16,17,18,19], and some studies suggest that these associations vary by the children’s age [20] and sex [21,22].

Understanding the associations between residing with someone with a SUD and pediatric mental health outcomes could inform prevention efforts [23]; however, research to date has been limited primarily to assessments of parent–child relationships (Table 1) [15,16,17,18,20,21,22,24,25,26,27,28]. Most of these studies found that living with a parent with a SUD increased children’s likelihood of having anxiety [15,16,17,20,24,25,26] and depression [15,16,17,18,20,24]. One qualitative study extends beyond parent–child relationships and supports the hypothesis that having a non-parent individual in the household with a SUD (e.g., a sibling) could be associated with children’s mental health [29]. However, no quantitative studies have examined the relationship between residing with anyone (regardless of relationship to the child) and children’s mental health outcomes. We therefore sought to assess whether residing with anyone who used substances was associated with the frequency of children’s anxiety and depression experience using a nationally representative sample. As a secondary objective, we stratified our analyses by children’s sex and age, respectively, to observe any differences based on these characteristics.

## 2. Methods

### 2.1. Study Population

We used 2019 National Health Interview Survey (NHIS) child interview data from the Centers for Disease Control and Prevention, National Center for Health Statistics [30]. The NHIS is a nationally representative, cross-sectional household survey that uses geographically clustered sampling techniques to identify individuals in all 50 states, as well as the District of Columbia [30]. The NHIS target population excludes people without a fixed household address, active duty military personnel living on military bases, people in long-term care facilities, people in correctional facilities, and U.S. nationals living in other countries [30]. In 2019, the NHIS performed 31,997 total household interviews, including 9193 interviews about randomly selected target children (each in separate households). Each selected household reported how many children (aged 0–17 years) lived in that household. If there was at least one child living in the selected household, the NHIS randomly selected one child from each household to be the target child. Survey respondents for the child portion of the survey were aged 18 years or older and were a parent, guardian, or other adult responsible for the target child’s well-being. Most household respondents (93.4%) were the target child’s parent (biological, adoptive, or stepparent), and the response rate was 59.1% [30]. All target children for the current analysis were 5–17 years of age (n = 6776). The Health Sciences Institutional Review Board considered this study “Not Human Subjects Research”.

### 2.2. Variable Definitions

The primary exposure variable of interest was the parent- or caregiver-reported answer to the question, “Did [the target child] ever live with anyone who had a problem with alcohol or drugs?” [31]. The answer choices to this question were yes, no, don’t know, or refused, and observations were omitted case-wise from the analysis if the respondent answered, “Don’t know” (n = 18) or “Refused” (n = 37) to this question, or if there were any missing data points for this question (n = 57). No information was available on the relationship between the child and the person who used substances, the length of time that the child resided with that person, or characteristics of the person who used substances.

We had two outcome variables of interest, and both were only assessed for target children who were 5–17 years of age. The first outcome variable was the parent- or caregiver-reported answer to the question, “How often does [the target child] seem very anxious, nervous, or worried? Would you say: daily, weekly, monthly, a few times a year, or never?” [31]. We excluded observations case-wise if the respondent answered, “Don’t know” (n = 9, 8 of whom had data for the exposure) or “Refused” (n = 12, 5 of whom had data for the exposure). The second outcome variable was the parent- or caregiver-reported answer to the question, “How often does [the target child] seem very sad or depressed? Would you say: daily, weekly, monthly, a few times a year, or never?” [31]. We excluded observations case-wise if the respondent answered, “Don’t know” (n = 7, 5 of whom had data for the exposure and anxiety question) or “Refused” (n = 11, none of whom had data for the exposure and anxiety question).

We chose covariates based on the existing literature [1,10,20,21,22,32,33] as those variables that were likely to affect both the exposure and outcome. A conceptual model of the hypothesized relationship among the covariates, exposure, and outcomes is represented in Supplementary Figure S1. The covariates included were the age of the child (in years) [20,32], the sex of the child (female or male) [21,22,32], the race/ethnicity of the child (Hispanic, non-Hispanic Asian, non-Hispanic Black, non-Hispanic White, or 2+ races/non-Hispanic other, considering the social construct of race/ethnicity as a proxy for structural racism) [1,10,32,33], annual household income (≤$34,999, $35,000–49,999, $50,000–74,999, $75,000–99,999, or ≥$100,000 (USD)) [1,10,32], and the highest education level of any adult in the household (less than high school diploma, high school diploma, some college or associate degree, or Bachelor’s degree or higher) [32]. Household was defined as an individual or group of two or more people residing together who are related by birth, marriage, or adoption, as well as any unrelated children who are cared for by the family and any unmarried cohabitating partners and their children [30]. We excluded observations case-wise if the included covariates were missing any data (n = 3 among participants with exposure and outcome data) or if the respondent specified “Refused” (n = 1 among participants with exposure and outcome data) for any of the included covariates. Our final analytic sample size was 6642.

### 2.3. Statistical Analysis

We used survey weighting in all analyses [34]. The survey weights were developed by the data distributor (NHIS) to account for the sampling method and non-response by invited individuals to help maximize the representativeness of the sample. The weights were based on regression models predicting the likelihood of responding to the survey and key health outcomes. Additionally, ranking procedures were used to calibrate the weights to population totals for age, sex, Hispanic or Latino origin and race, educational attainment, Census division, and Metropolitan Statistical Area status. We first examined univariate descriptive statistics for each variable to assess measures of central tendency and the overall distribution of each variable. Then, we used an adjusted Wald test and design-based F tests to compare demographic characteristics of children with and without a history of residence with someone who used substances. We used the same tests to compare demographic and mental health characteristics of children included and excluded from the analysis. Next, we estimated unadjusted (covariates not included) and adjusted (covariates included) multinomial logistic regression models for the association between having ever resided with someone who used substances and children’s anxiety (five categories (never, a few times a year, monthly, weekly, daily); referent = never anxious) and between having ever resided with someone who used substances and children’s depression (same five frequency categories; referent = never depressed). All the primary results reported refer to the adjusted associations. Covariates in adjusted models included children’s age, children’s sex, children’s race/ethnicity, household income, and highest education level of any adult in the household (primary models). To assess differences by children’s age, we estimated age-stratified (young children 5–11 years/adolescents 12–17 years) [35] multinomial regression models adjusted for the same set of covariates as the primary models (excluding children’s age). Similarly, to assess differences by children’s sex, we estimated sex-stratified multinomial regression models adjusted for the same set of covariates as the primary models (excluding children’s sex). Within each stratum, we considered trends to be different by age or sex if the point estimates for one group were not included in the 95% confidence interval for the other group. The effect estimates for each model are relative risk ratios, interpreted equivalently to odds ratios in logistic regression [36]. In each multivariable model, we assessed collinearity by examining the variance inflation factor (with a cut-off limit of 5), we assessed leverage by plotting predicted values against the leverage statistic, and we assessed influential points by plotting predicted probabilities against the Pregibon influential point statistic. All analyses were conducted in Stata SE v16.1 (StataCorp LLC; College Station, TX, USA).

## 3. Results

Table 2 describes the sample characteristics. Overall, we found that 9.7% of children had lived with someone who used substances. Residing with someone who used substances was not significantly associated with children’s sex (*F* = 0.43, *p* = 0.514) but was significantly associated with children’s anxiety frequency (*F* = 25.54, *p* < 0.001), children’s depression frequency (*F* = 43.26, *p* < 0.001), children’s age (*F* = 24.24, *p* < 0.001), children’s race/ethnicity (*F* = 19.62, *p* < 0.001), annual household income (*F* = 13.39, *p* < 0.001), and highest education level of any adult in the household (*F* = 16.87, *p* < 0.001). Table 3 describes the characteristics of those included and excluded from the analysis. Whether or not a target child in the NHIS was included or excluded from the present analysis was not significantly associated with the reported frequency of children’s anxiety (*F* = 0.69, *p* = 0.588), reported frequency of children’s depression (*F* = 1.51, *p* = 0.201), children’s sex (*F* < 0.01, *p* = 0.978), children’s race/ethnicity (*F* = 0.71, *p* = 0.585), or the highest education level of any adult in the household (*F* = 0.82, *p* = 0.479); however, it was associated with having ever lived with someone who used substances (*F* = 68.22, *p* < 0.001) and annual household income (*F* = 5.00, *p* = 0.001). Additionally, excluded children were significantly younger (*p* < 0.001) because the questions pertaining to children’s mental health were not asked for children aged 0–4 years (mean age of excluded children = 2.5 years).

**Figure 1:**
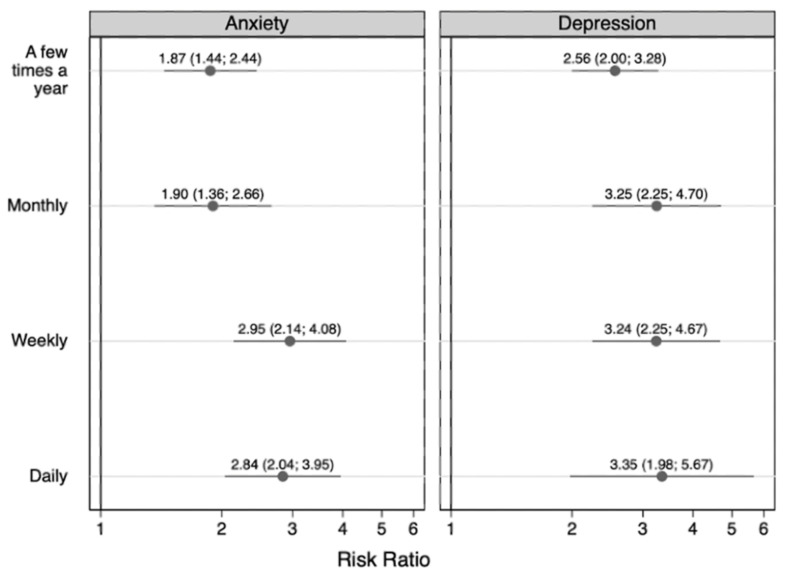
The association between having ever resided with someone with a substance use disorder and frequency of children’s anxiety and depression (n = 6642). Symbols indicate relative risk ratios and lines indicate 95% confidence intervals. The referent groups are children who never experienced anxiety and children who never experienced depression.

Figure 1 shows the primary multinomial logistic regression model results.

The full results from the primary multinomial logistic models are presented in Table 4 (outcome = children’s anxiety) and Table 5 (outcome = children’s depression). Compared to children who had never lived with someone who used substances, children who had lived with someone who used substances were significantly more likely to experience anxiety (*F* = 17.24, *p* < 0.001) and depression (*F* = 24.59, *p* < 0.001) at each frequency interval. Specifically, they were more likely to experience weekly (risk ratio (RR) = 2.95; 95% confidence interval (CI) = 2.14, 4.08) or daily (RR = 2.84; 95% CI = 2.04, 3.95) anxiety, as well as weekly (RR = 3.24; 95% CI = 2.25, 4.67) or daily (RR = 3.35; 95% CI = 1.98, 5.67) depression.

**Figure 2:**
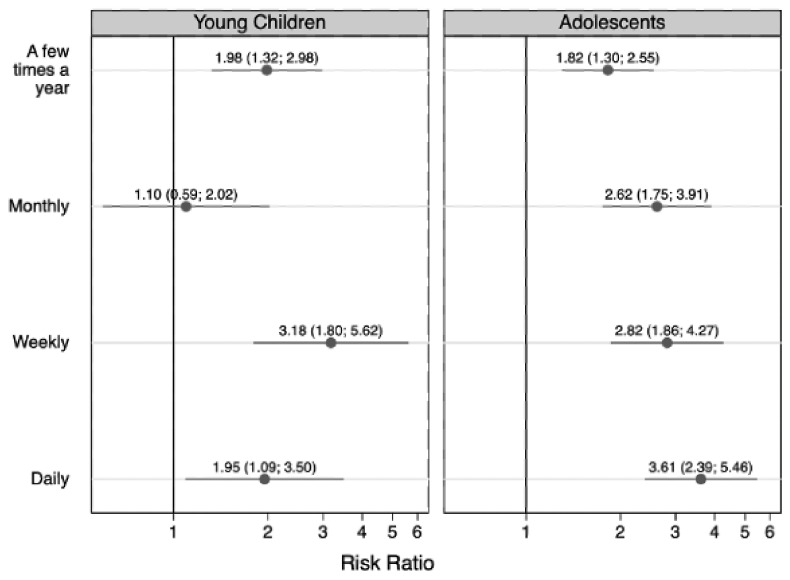
The age-stratified association between having ever resided with someone with a substance use disorder and frequency of children’s anxiety (young children [5–11 years of age] n = 3135; adolescents [12–17 years of age] n = 3507). Symbols indicate relative risk ratios and lines indicate 95% confidence intervals. The referent group is children who never experienced anxiety.

**Figure 3:**
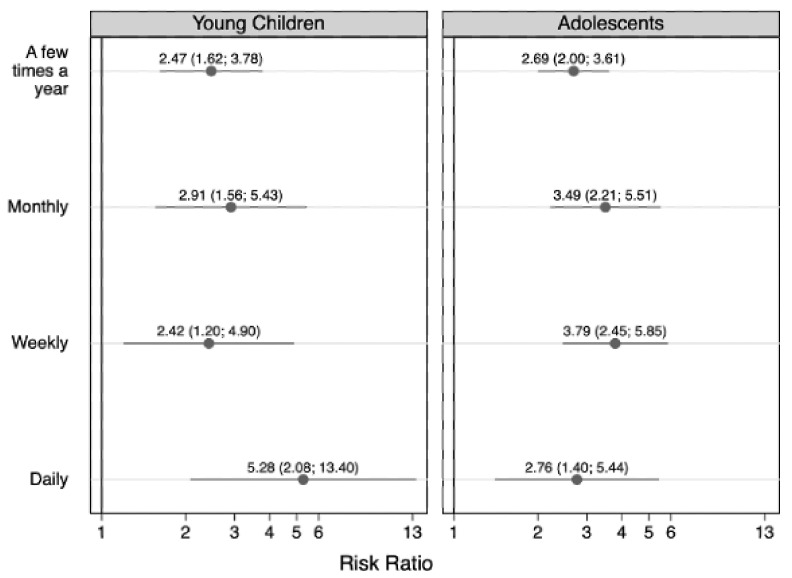
The age-stratified association between having ever resided with someone with a substance use disorder and frequency of children’s depression (young children [5–11 years of age] n = 3135; adolescents [12–17 years of age] n = 3507). Symbols indicate relative risk ratios and lines indicate 95% confidence intervals. The referent group is children who never experienced depression.

**Figure 4:**
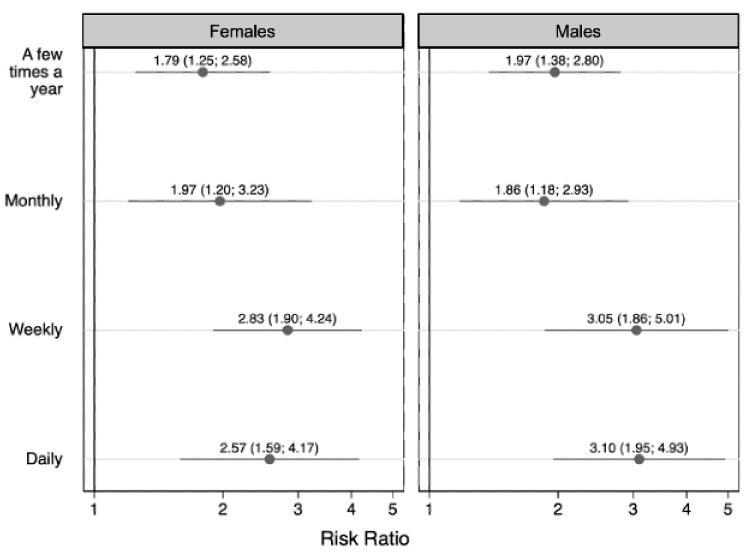
The sex-stratified association between having ever resided with someone with a substance use disorder and frequency of children’s anxiety (females n = 3201; males n = 3441). Symbols indicate relative risk ratios and lines indicate 95% confidence intervals. The referent group is children who never experienced anxiety.

**Figure 5:**
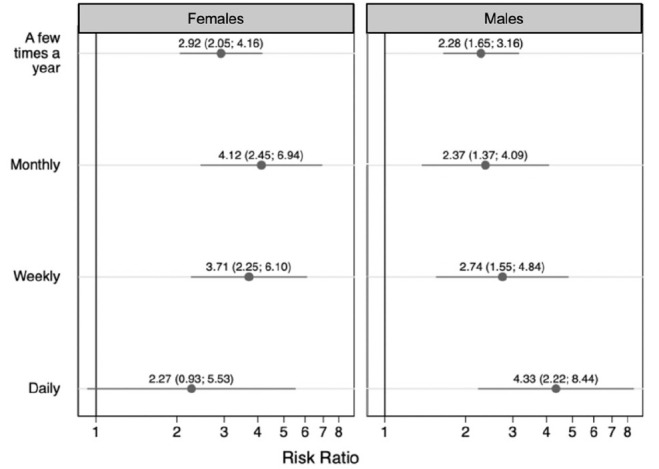
The sex-stratified association between having ever resided with someone with a substance use disorder and frequency of children’s depression (females n = 3201; males n = 3441). Symbols indicate relative risk ratios and lines indicate 95% confidence intervals. The referent group is children who never experienced depression.

Figure 2, Figure 3, Figure 4 and Figure 5 show the results from the age- and sex-stratified analyses. The associations between residence with someone who used substances and the frequency of anxiety (Figure 2) and depression (Figure 3) differed based on the children’s age. Associations with monthly and daily anxiety were stronger among adolescents (monthly anxiety: RR = 2.62 (95% CI: 1.75, 3.91), daily anxiety: RR = 3.61 (95% CI: 2.39, 5.46)) than younger children (monthly anxiety: RR = 1.10 (95% CI: 0.59, 2.02), daily anxiety: RR = 1.95 (95% CI: 1.09, 3.50)), as is shown by the mostly non-overlapping confidence intervals for these frequencies of anxiety compared to the referent (never anxious; Figure 2). Associations with weekly depression were marginally stronger among adolescents (weekly depression: RR = 3.79 (95% CI: 2.45, 5.85)) than younger children (weekly depression: RR = 2.42 (95% CI: 1.20, 4.90)), and associations with daily depression were marginally stronger among younger children (daily depression: RR = 5.28 (95% CI: 2.08, 13.40)) than adolescents (daily depression: RR = 2.76 (95% CI: 1.40, 5.44)) (Figure 3), but these trends should be interpreted cautiously considering the small sample sizes (Table 6).

In the sex-stratified models, we did not observe differences in the association between residence with someone who used substances and anxiety frequency between males and females, as is shown by the overlapping confidence intervals between the results for males and females (Figure 4), but we did observe evidence that the relationship with depression frequency differed by sex (Figure 5). The association between residence with someone who used substances and monthly depression was stronger among females (monthly depression: RR = 4.12 (95% CI: 2.45, 6.94)) than males (monthly depression: RR = 2.37 (95% CI: 1.37, 4.09)), whereas the association between residence with someone who used substances and daily depression was marginally stronger among males (daily depression: RR = 4.33 (95% CI: 2.22, 8.44)) than females (daily depression: RR = 2.27 (95% CI: 0.93, 5.53)) (Figure 5). As with the age-stratified results, the sex-stratified associations with daily depression should be interpreted cautiously considering the small sample sizes (Table 6).

## 4. Discussion

Our primary finding was that if a child had ever lived with someone who used substances, their likelihood of experiencing anxiety and/or depression on a weekly or daily basis was significantly increased (*p* < 0.05). The associations with depression were slightly stronger than the associations with anxiety. Our secondary finding was that associations with both anxiety and depression frequency differed based on age, and that associations with depression differed by sex. These observations address a gap in the literature, as there have been few studies to date [29] exploring how residing with anyone who used substances affects the experience of childhood anxiety and depression. We also found that the likelihood of living with someone who used substances differed by children’s age, annual household income, highest educational attainment by an adult in the household, and other factors, and these findings add to the literature by helping identify potential screening and intervention factors (e.g., perhaps improving the social safety net could help reduce the likelihood of children residing with someone who uses substances). Furthermore, we provide the first national estimate for the proportion of children in the U.S. who live with, or have ever lived with, any person who used substances (9.7%). Our results suggest that screening children for anxiety, depression, and for residence with someone who uses substances could promote positive child development and present an opportunity for assisting the person who uses substances.

Our primary results were largely consistent with studies that examined the relationship between residence with a parent with a SUD and the likelihood of having mental health disorders in children (Table 1). The majority of these studies observed a significantly increased likelihood of internalizing disorders, such as anxiety [15,16,17,20,24,25,26] and depression [15,16,17,18,20,24], among offspring who had at least one parent with alcohol use disorder and/or SUD. Furthermore, the magnitude of our effect estimates for daily anxiety (RR = 2.84; 95% CI: 2.04, 3.95; referent = never anxious) and daily depression (RR = 3.35; 95% CI: 1.98, 5.67; referent = never depressed) are comparable to the effect estimates obtained from other studies examining the prevalence of mental health disorders among children with at least one parent with a SUD, compared to children without a parent with a SUD. For example, Chassin et al. obtained an OR of 2.40 (95% CI: 1.40, 4.10) for a depression diagnosis and an OR of 1.55 (95% CI: 0.96, 2.27) for an anxiety disorder diagnosis [22]. Similarly, Clark et al. obtained an OR of 2.10 (95% CI: 1.40, 3.00) for a depression diagnosis and an OR of 1.90 (95% CI: 1.30, 2.80) for an anxiety diagnosis [16]. Finally, Hill et al. obtained an OR of 4.40 (95% CI: 1.72, 11.26) for a depression diagnosis [20]. Our analysis builds on previous work by suggesting that the associations between residence with someone who used substances and children’s anxiety and depression experiences may not be limited to the parent–child relationship (i.e., residing with *anyone* who used substances was associated with increased frequency of children’s anxiety and depression). Additionally, our analysis builds on previous literature suggesting that sociodemographic characteristics (e.g., children’s age, children’s sex, children’s race/ethnicity, annual household income) are associated with the frequency of children’s anxiety and depression [1,10,20,21,22,32,33].

Furthermore, we added to the literature examining whether associations between exposure to people in the household who used substances and children’s mental health experiences differ based on age. Our finding that associations with more frequent anxiety were stronger among adolescents than younger children makes sense considering existing literature suggesting that age is an effect modifier of the relationship between having a parent with a SUD and children’s mental health outcomes [20]. These trends may be partially explained by the fact that adolescents are at higher risk of having anxiety and depression than younger children [37,38]. Additionally, due to their age, adolescents have had more years of possible exposure (i.e., have lived with someone who used substances for a longer period of time) than younger children, which could result in a higher risk of anxiety and depression (or at least a greater likelihood of having these conditions identified). Our results for how age modified associations with depression were less consistent overall and with previous studies, perhaps because we had an insufficient sample size to adequately assess age-stratified associations with depression.

We also examined whether associations between residing with someone who used substances and children’s mental health experiences differed by sex. Previous studies suggest that sex differences may exist, though the evidence from these studies has been limited by very small sample sizes. For example, one study suggested that the likelihood of anxiety disorders was higher for males who had a parent with a SUD (10%, n = 40) than for males who did not (3.9%, n = 26) and lower for females who had a parent with a SUD (10.8%, n = 37) than for females who did not (16.1%, n = 31) [21]. Another suggested that certain pathways from parental alcohol use disorders to adolescent internalizing symptoms to young adult outcomes were sex-dependent [22]. A third study indicated that associations with depression (but not anxiety) may have been somewhat stronger in males than females (though even in males, the relationships were not statistically significant and the associations were only adjusted for age) [28]. Consistent with this study, we observed some evidence for sex-specific associations with depression but not anxiety. Our work adds to the literature as it is the largest study to date examining sex-specific associations and the first examining associations with anxiety and depression frequency.

We found that residence with any household member who used substances is associated with a higher frequency of children’s anxiety and depression experiences. This finding is consistent with the literature, which suggests that the home life of individuals with a SUD is often more financially stressful, prone to law enforcement involvement, unstable, associated with worse parent–child relationships, and affected by structural racism [3,4,6,9,13,15,39,40]. These adverse experiences can contribute to internalizing disorders, such as anxiety and depression [8,9,10,11,13,15,39]. Furthermore, children learn how to regulate their affect—the ways in which one expresses and handles emotions—from their parents/caregivers [9], and oftentimes parents/caregivers with a SUD do not themselves have a regulated affect, a circumstance which might have resulted from (or led to) their disorder [9,15]. As a consequence, their children may be less likely to develop a healthy affect regulation or to be presented an opportunity to engage with mental health professionals [39], leading to an increased occurrence of internalizing disorders [9,15,39]. Our analysis supports these observations and suggests that the home environment of someone who uses substances, regardless of the relationship of the person who uses substances to the child, may be associated with children’s mental health. Our analysis also suggests a plausible explanation for previous research indicating that genetic variability alone does not explain current trends in mental health occurrence [41].

Our study had several strengths and limitations. The primary strength was the nationally representative and large sample with individual-level data for a number of key covariates. Although selection bias was not a major concern as response rates were unlikely to depend on children’s anxiety and depression levels, and survey weighting should help account for differences in response rates by different sociodemographic factors, it is still possible that selection bias was introduced due to our inclusion criteria for the analysis (e.g., the sample excluded differed from the sample included in the likelihood of residing with someone who used substances). Similarly, recall bias, social desirability bias, and other differential exposure misclassification concerns may be less problematic in our study than in other cross-sectional studies examining the associations between residence with someone who used substances and children’s anxiety and depression since respondents were not asked to identify characteristics of *who* used substances. However, given the sensitive nature of SUD and children’s mental health concerns, we cannot entirely rule out social desirability bias. If true, we might expect respondents to underreport both exposure and outcomes in our study, thus resulting in an underestimate of the true association. Additionally, since we do not know the relationship between the child and the person who used substances, we cannot draw conclusions about the types of relationships that may be most strongly associated with children’s anxiety and depression. Furthermore, considering we do not know when the child was exposed to living with someone who used substances or for how long, we cannot comment on how duration of exposure affects children’s anxiety and depression. Similarly, we do not have any clinician measures of anxiety or depression, a potential limitation to the robustness of the outcome measures. Finally, considering the cross-sectional nature of the study, we cannot draw any causal conclusions and we cannot comment on the temporal relationship between exposure and outcomes.

Future work should explore how specific relationships, and how residence for various durations of time, affect the associations. Similarly, future work could explore how robust the associations are to different measures of exposure and outcome (e.g., how clinician-diagnosed measures might compare to self-reported measures). Additionally, future work should examine in more detail how sociodemographic variables, such as children’s race/ethnicity, annual household income, and highest educational attainment by an adult in the household, are associated with residing with someone who used substances and with children’s anxiety and depression experiences. Finally, future longitudinal studies should examine the associations between residence of individuals with different relationships to the child and the incidence and severity of children’s anxiety and depression, as we were unable to incorporate this information in our cross-sectional data.

## 5. Conclusions

Our nationally representative study suggests that residing with someone who used substances is associated with the frequency of children’s anxiety and depression experiences. This observation can help inform prevention, screening, and treatment opportunities for the individual who used substances and the child experiencing anxiety and/or depression. Childhood and adolescence are the most vulnerable times for developing and manifesting mental health disorders [42], and early screening and treatment strategies can greatly enhance the chances for those children to become healthy adults [23], while also offering potential improved opportunities for relationship building in the home. Ultimately, we hope that our findings can help enhance screening and prevention efforts for children who might be experiencing anxiety and/or depression, and for children who might be residing with someone who uses substances. Additionally, these screening efforts could help the person using substances receive the care they need.

## Figures and Tables

**Table 1:** Studies that have examined the relationship between residence with a parent with a substance use disorder (SUD) or alcohol disorder (AD) and anxiety and depression risk among their children.

Study	Parent(s)	Children		
Authors	Diagnosis	n (% Male)	Age Range at Baseline (Years)	Outcome(s) *
Bountress and Chassin [15]	SUD	567 (54.4)	6–13	internalizing behavior (including anxiety/depression) **
Chassin et al. [22]	AD	407 (53.1)	10–15	internalizing symptoms ∞
Clark et al. [16]	SUD	1167 (62.0)	6–14	Anxiety ** Depression **
Díaz et al. [17]	AD	518 (50.2)	6–17	Anxiety ** Depression **
Hill et al. [20]	AD	378 (not specified)	8–18	internalizing disorders (including anxiety/depression) ** ^ depression ** ^
Hill and Hruska [27]	AD	95 (52.6)	8–18	anxiety depression
Kuperman et al. [26]	AD	463 (not specified)	(not specified)	anxiety (only separation anxiety) **
Merikangas et al. [21]	SUD	192 (51.0)	7–18	Anxiety ∞ depression
Nunes et al. [28]	SUD	209 (50.7)	6–17	anxiety depression
Reich et al. [25]	AD	158 (54.2)	6–18	Anxiety ** depression
Vidal et al. [18]	SUD	276 (52.9)	6–17	anxiety depression **
Wilens et al. [24]	SUD	183 (58.5)	6–18	Anxiety ** Depression **

* Here, we only include anxiety and depression even if the original study included additional outcomes. ** Outcome was found to be significantly associated with having a parent/parents with a SUD or AD. ^ Stronger associations were observed for adolescents than for younger children. ∞ The study found evidence for effect modification by sex.

**Table 2:** Descriptive characteristics of the sample children—2019 National Health Interview Survey (n = 6642) *.

Characteristic	Total n (%)	Has Lived with Someone Who Used Substances n (%)	Has Not Lived with Someone Who Used Substances n (%)
**Sample size**	6642 (100.0)	706 (9.7)	5936 (90.3)
Anxiety frequency **			
never	3718 (57.1)	265 (38.2)	3453 (59.1)
a few times a year	1521 (22.7)	181 (27.2)	1340 (22.2)
monthly	481 (6.7)	80 (9.0)	401 (6.5)
weekly	560 (8.3)	104 (15.5)	456 (7.6)
daily	362 (5.3)	76 (10.2)	286 (4.7)
Depression frequency **			
never	4843 (74.7)	353 (51.2)	4490 (77.2)
a few times a year	1126 (16.0)	187 (27.2)	939 (14.8)
monthly	296 (4.2)	70 (8.8)	226 (3.7)
weekly	263 (3.7)	68 (8.8)	195 (3.1)
daily	114 (1.5)	28 (4.0)	86 (1.3)
Age ** *(years)*			
*Minimum–maximum*	5–17	5–17	5–17
*mean (95% CI)*	11.1 (10.9–11.2)	11.9 (11.5–12.2)	11.0 (10.8–11.1)
**Sex**			
female	3201 (48.9)	342 (50.4)	2859 (48.8)
male	3441 (51.1)	364 (49.6)	3077 (51.2)
Race/ethnicity **			
Hispanic	1567 (25.4)	130 (19.5)	1437 (26.1)
non-Hispanic Asian	366 (4.3)	3 (0.4)	363 (4.8)
non-Hispanic Black	766 (12.8)	41 (6.0)	725 (13.5)
non-Hispanic White	3561 (52.1)	479 (66.0)	3082 (50.6)
2+ races/non-Hispanic other	382 (5.3)	53 (8.1)	329 (5.0)
Annual household income (USD) **			
$0–34,999	1381 (22.4)	210 (30.9)	1171 (21.5)
$35,000–49,999	770 (11.9)	114 (16.2)	656 (11.4)
$50,000–74,999	1127 (16.8)	124 (18.2)	1003 (16.6)
$75,000–99,999	871 (12.5)	96 (11.6)	775 (12.6)
$100,000 or greater	2493 (36.4)	162 (23.1)	2331 (37.8)
Highest educational attainment by an adult in the household **			
less than high school diploma	454 (8.7)	62 (10.2)	392 (8.6)
high school diploma	1046 (16.0)	128 (17.2)	918 (15.9)
some college/associate degree	1999 (29.1)	290 (41.5)	1709 (27.8)
college degree or greater	3143 (46.2)	226 (31.1)	2917 (47.8)

* Cell counts are unweighted, percentages and means are weighted. ** Variable is significantly (*p* < 0.05) associated with having lived with someone who used substances.

**Table 3:** Descriptive characteristics of those included and excluded from the analysis—2019 National Health Interview Survey *.

Characteristic	Total n (%)	Included in Analysis n (%)	Excluded from Analysis n (%) ***
Lived with someone who used substances **			
no	8275 (92.0)	5936 (90.3)	2339 (96.5)
yes	789 (8.0)	706 (9.7)	83 (3.5)
*total*	9064 (100.0)	6642 (100.0)	2422 (100.0)
**Anxiety frequency**			
never	3785 (57.2)	3718 (57.1)	67 (62.8)
a few times a year	1545 (22.6)	1521 (22.7)	24 (2.1)
monthly	490 (6.7)	481 (6.7)	9 (7.4)
weekly	566 (8.2)	560 (8.3)	6 (4.3)
daily	369 (5.2)	362 (5.3)	7 (4.0)
*total*	6755 (100.0)	6642 (100.0)	113 (100.0)
**Depression frequency**			
never	4933 (74.8)	4843 (74.7)	90 (80.6)
a few times a year	1144 (15.9)	1126 (16.0)	18 (13.8)
monthly	297 (4.1)	296 (4.2)	1 (0.6)
weekly	270 (3.7)	263 (3.7)	7 (5.0)
daily	114 (1.5)	114 (1.5)	0 (0.0)
*total*	6758 (100.0)	6642 (100.0)	116 (100.0)
Age ** *(years)*			
*minimum*–*maximum*	0–17	5–17	0–17
*mean (95% CI)*	8.6 (8.5–8.7)	11.1 (10.9–11.2)	2.5 (2.4–2.6)
*total*	9193 (100.0)	6642 (100.0)	2551 (100.0)
**Sex**			
female	4484 (49.0)	3201 (48.9)	1283 (49.0)
male	4705 (51.1)	3441 (51.1)	1264 (51.0)
*total*	9189 (100.0)	6642 (100.0)	2547 (100.0)
**Race/ethnicity**			
Hispanic	2173 (25.7)	1567 (25.4)	606 (26.4)
non-Hispanic Asian	511 (4.4)	366 (4.3)	145 (4.5)
non-Hispanic Black	1022 (12.7)	766 (12.8)	256 (12.6)
non-Hispanic White	4921 (51.6)	3561 (52.1)	1360 (50.4)
2+ races/non-Hispanic other	566 (5.6)	382 (5.3)	184 (6.2)
*total*	9193 (100.0)	6642 (100.0)	2551 (100.0)
Annual household income (USD) **			
$0–34,999	1921 (22.8)	1381 (22.4)	540 (23.9)
$35,000–49,999	1079 (12.1)	770 (11.9)	309 (12.5)
$50,000–74,999	1585 (17.4)	1127 (16.8)	458 (19.1)
$75,000–99,999	1257 (13.0)	871 (12.5)	386 (14.1)
$100,000 or greater	3351 (34.7)	2493 (36.4)	858 (30.4)
*total*	9193 (100.0)	6642 (100.0)	2551 (100.0)
**Highest educational attainment by an adult in the household**			
less than high school diploma	610 (8.5)	454 (8.7)	156 (8.1)
high school diploma	1451 (16.4)	1046 (16.0)	405 (17.4)
some college/associate degree	2693 (28.8)	1999 (29.1)	694 (27.9)
college degree or greater	4426 (46.3)	3143 (46.2)	1283 (46.7)
*total*	9180 (100.0)	6642 (100.0)	2538 (100.0)

* Cell counts are unweighted, percentages and means are weighted. ** Variable is significantly (*p* < 0.05) associated with being included or excluded from the analysis. *** We excluded observations case-wise if there were any missing data for exposures, outcomes, or covariates. Responses of “don’t know” for outcomes were also excluded. Additionally, of the 2422 excluded observations, 2288 were excluded due to the target child being younger than 5 years of age, meaning that there were no data for the outcomes for these observations.

**Table 4:** Multinomial regression models for the association between having ever resided with someone who used substances and the frequency of children’s anxiety (referent = never experienced anxiety)—2019 National Health Interview Survey (n = 6642).

Covariate	A Few Times a Year	Monthly	Weekly	Daily	*F*-Statistic
	RR (95% CI) **	RR (95% CI)	RR (95% CI)	RR (95% CI)	
**Lived with someone who used substances**					17.24 *
no (*reference*)	1.0 (referent)	1.0 (referent)	1.0 (referent)	1.0 (referent)	
yes	1.87 (1.44, 2.44)	1.90 (1.36, 2.66)	2.95 (2.14, 4.08)	2.84 (2.04, 3.95)	
**Age**					9.24 *
	1.05 (1.03, 1.06)	1.07 (1.04, 1.11)	1.04 (1.01, 1.07)	1.05 (1.01, 1.09)	
**Sex**					4.09 *
female (*reference*)	1.0 (referent)	1.0 (referent)	1.0 (referent)	1.0 (referent)	
male	0.85 (0.74, 0.97)	0.80 (0.64, 0.99)	0.70 (0.56, 0.87)	1.00 (0.77, 1.29)	
**Race/ethnicity**					6.16 *
non-Hispanic White (*reference*)	1.0 (referent)	1.0 (referent)	1.0 (referent)	1.0 (referent)	
Hispanic	0.70 (0.58, 0.84)	0.54 (0.38, 0.76)	0.52 (0.37, 0.73)	0.67 (0.49, 0.94)	
non-Hispanic Asian	0.77 (0.57, 1.04)	0.24 (0.10, 0.54)	0.26 (0.14, 0.49)	0.31 (0.13, 0.75)	
non-Hispanic Black	0.60 (0.46, 0.78)	0.26 (0.15, 0.44)	0.32 (0.21, 0.49)	0.62 (0.39, 0.96)	
2+ races/non-Hispanic other	0.66 (0.46, 0.94)	0.65 (0.40, 1.05)	0.68 (0.42, 1.10)	0.82 (0.50, 1.35)	
**Annual household income (USD)**					2.57 *
$0–34,999 (*reference*)	1.0 (referent)	1.0 (referent)	1.0 (referent)	1.0 (referent)	
$35,000–49,999	1.28 (0.97, 1.69)	0.84 (0.56, 1.25)	1.15 (0.74, 1.80)	0.94 (0.60, 1.49)	
$50,000–74,999	1.14 (0.90, 1.45)	0.55 (0.37, 0.81)	1.13 (0.73, 1.73)	0.52 (0.33, 0.84)	
$75,000–99,999	1.66 (1.27, 2.18)	0.89 (0.56, 1.42)	1.40 (0.85, 2.30)	0.97 (0.62, 1.52)	
$100,000 or greater	1.52 (1.20, 1.91)	0.97 (0.68, 1.39)	1.39 (0.93, 2.08)	0.65 (0.43, 0.99)	
**Highest educational attainment by an adult in the household**					2.41 *
less than high school diploma (*reference*)	1.0 (referent)	1.0 (referent)	1.0 (referent)	1.0 (referent)	
high school diploma	1.32 (0.90, 1.94)	2.21 (1.05, 4.64)	1.63 (0.85, 3.10)	0.59 (0.35, 1.00)	
some college/associate degree	1.62 (1.11, 2.37)	2.77 (1.42, 5.42)	1.76 (0.92, 3.37)	0.71 (0.43, 1.18)	
college degree or greater	1.63 (1.13, 2.35)	3.26 (1.62, 6.58)	2.02 (1.03, 3.94)	0.85 (0.49, 1.46)	

* Variable is significantly (*p* < 0.05) associated with the frequency of children’s anxiety. ** RR = relative risk ratios (interpreted equivalently to odds ratios in logistic regression), 95% CI = 95% confidence interval.

**Table 5:** Multinomial regression models for the association between having ever resided with someone who used substances and the frequency of children’s depression (referent = never experienced depression)—2019 National Health Interview Survey (n = 6642).

Covariate	A Few Times a Year	Monthly	Weekly	Daily	*F*-Statistic
	RR (95% CI) **	RR (95% CI)	RR (95% CI)	RR (95% CI)	
**Lived with someone who used substances**					24.59 *
no (*reference*)	1.0 (referent)	1.0 (referent)	1.0 (referent)	1.0 (referent)	
yes	2.56 (2.00, 3.28)	3.25 (2.25, 4.70)	3.24 (2.25, 4.67)	3.35 (1.98, 5.67)	
**Age**					32.38 *
	1.11 (1.09, 1.13)	1.13 (1.09, 1.17)	1.11 (1.06, 1.15)	1.16 (1.08, 1.24)	
**Sex**					5.12 *
female (*reference*)	1.0 (referent)	1.0 (referent)	1.0 (referent)	1.0 (referent)	
male	0.81 (0.69, 0.95)	0.70 (0.53, 0.91)	0.59 (0.43, 0.82)	0.97 (0.61, 1.52)	
**Race/ethnicity**					3.83 *
non-Hispanic White (*reference*)	1.0 (referent)	1.0 (referent)	1.0 (referent)	1.0 (referent)	
Hispanic	0.69 (0.55, 0.86)	0.46 (0.31, 0.69)	0.52 (0.34, 0.77)	0.55 (0.31, 0.97)	
non-Hispanic Asian	0.68 (0.45, 1.00)	0.27 (0.12, 0.62)	0.19 (0.07, 0.49)	0.23 (0.05, 0.97)	
non-Hispanic Black	0.75 (0.56, 0.99)	0.53 (0.33, 0.86)	0.48 (0.27, 0.84)	0.58 (0.27, 1.23)	
2+ races/non-Hispanic other	1.09 (0.78, 1.53)	0.80 (0.39, 1.64)	0.95 (0.55, 1.63)	1.18 (0.57, 2.44)	
**Annual household income (USD)**					2.18 *
$0–$34,999 (*reference*)	1.0 (referent)	1.0 (referent)	1.0 (referent)	1.0 (referent)	
$35,000–$49,999	1.06 (0.79, 1.43)	1.30 (0.76, 2.25)	0.79 (0.45, 1.39)	0.54 (0.26, 1.11)	
$50,000–$74,999	0.95 (0.72, 1.26)	1.06 (0.63, 1.78)	0.42 (0.25, 0.71)	0.32 (0.15, 0.69)	
$75,000–$99,999	1.14 (0.82, 1.57)	1.09 (0.63, 1.90)	0.44 (0.25, 0.77)	0.71 (0.38, 1.34)	
$100,000 or greater	1.03 (0.79, 1.34)	1.45 (0.92, 2.29)	0.44 (0.27, 0.73)	0.34 (0.16, 0.75)	
**Highest educational attainment by an adult in the household**					1.55
less than high school diploma (*reference*)	1.0 (referent)	1.0 (referent)	1.0 (referent)	1.0 (referent)	
high school diploma	1.01 (0.68, 1.51)	1.84 (0.91, 3.71)	0.88 (0.46, 1.71)	0.61 (0.28, 1.30)	
some college/associate degree	0.93 (0.64, 1.35)	1.32 (0.67, 2.59)	1.29 (0.70, 2.39)	0.41 (0.20, 0.83)	
college degree or greater	1.08 (0.74, 1.57)	1.45 (0.72, 2.91)	1.46 (0.77, 2.78)	0.35 (0.15, 0.81)	

* Variable is significantly (*p* < 0.05) associated with the frequency of children’s depression. ** RR = relative risk ratios (interpreted equivalently to odds ratios in logistic regression), 95% CI = 95% confidence interval.

**Table 6:** Sample size for stratified analyses—2019 National Health Interview Survey (n = 6642) *.

Characteristic	Children’s Age		Children’s Sex	
	5–11 Years n (%)	12–17 Years n (%)	Females n (%)	Males n (%)
**Total**	3135 (100.0)	3507 (100.0)	3201 (100.0)	3441 (100.0)
**Anxiety**				
never	1904 (60.6)	1814 (53.2)	1701 (54.7)	2017 (59.3)
a few times a year	648 (20.9)	873 (24.6)	751 (23.6)	770 (21.8)
monthly	206 (5.9)	275 (7.6)	249 (7.2)	232 (6.2)
weekly	233 (7.8)	327 (8.8)	313 (9.5)	247 (7.2)
daily	144 (4.8)	218 (5.8)	187 (5.0)	175 (5.5)
**Depression**				
never	2504 (80.6)	2339 (68.1)	2252 (72.2)	2591 (77.1)
a few times a year	414 (12.6)	712 (19.8)	573 (17.1)	553 (14.9)
monthly	99 (3.0)	197 (5.4)	159 (4.8)	137 (3.6)
weekly	85 (2.9)	178 (4.5)	160 (4.5)	103 (2.9)
daily	33 (0.9)	81 (2.2)	57 (1.5)	57 (1.6)

* Cell counts are unweighted, percentages and means are weighted.

## Data Availability

Publicly available data were analyzed in this study. Data can be found here: https://www.cdc.gov/nchs/nhis/2019nhis.htm (accessed on 7 July 2022).

## Supplementary Materials

The following supporting information can be downloaded at: https://www.mdpi.com/article/10.3390/children9091296/s1, Figure S1: The conceptual model shows hypothesized relationships among covariates, exposure, and outcomes. For simplicity, arrows are used directly from each covariate to the exposure and outcome, even if the true underlying relationship may be more indirect. *Race/ethnicity of child is used as a covariate to serve as a proxy for racism-related barriers.
