# Supplementary material for: Residence with a Person Who Used Substances and Childhood Anxiety and Depression: A Cross-Sectional Analysis of the 2019 National Health Interview Survey
Source: Children (Basel). 2022 Aug 26;9(9):1296. doi: 10.3390/children9091296 (PMC9497714; doi:10.3390/children9091296)
Supplement: Supplementary file 1 [file children-09-01296-s001.zip › children-1831202-SI.pdf]

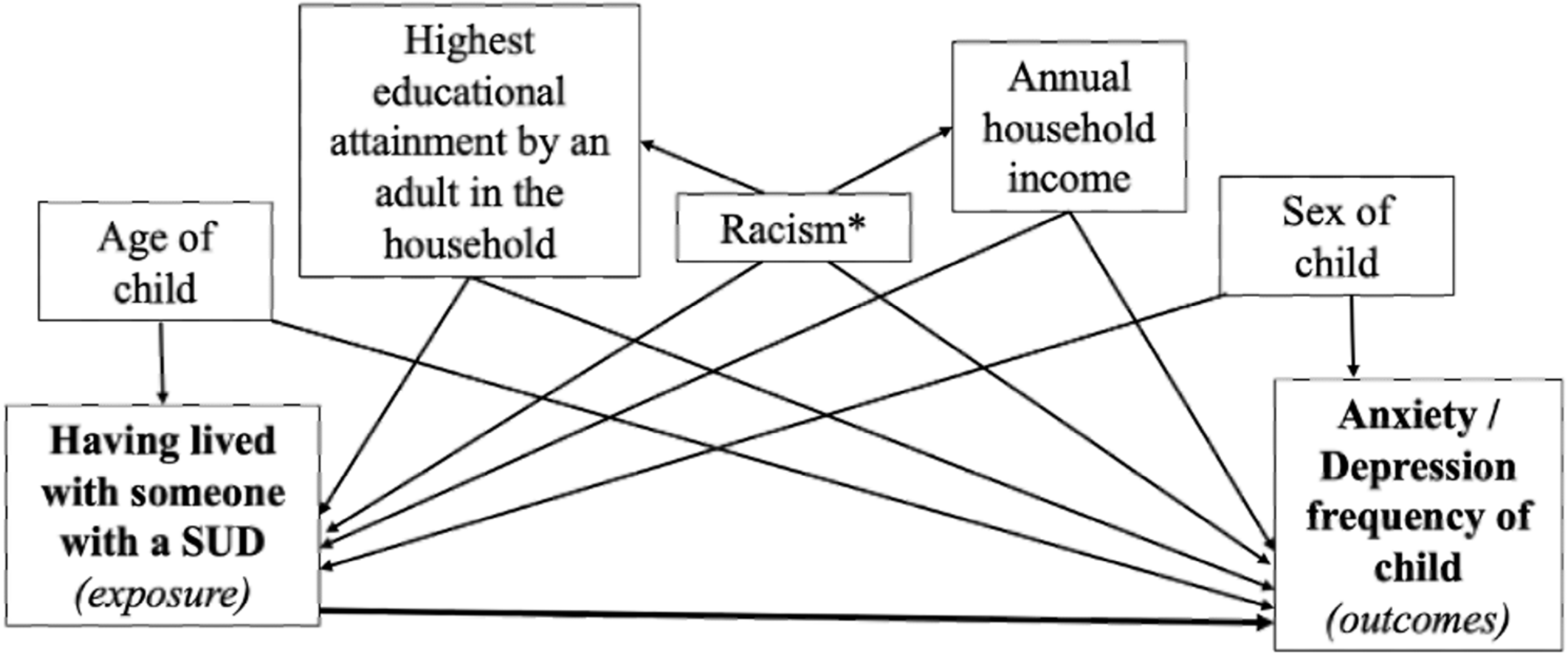

Supplemental Figure S1. The conceptual model shows hypothesized relationships among covariates, exposure, and outcomes. For simplicity, arrows are used directly from each covariate to the exposure and outcome, even if the true underlying relationship may be more indirect. *Race/ethnicity of child is used as a covariate to serve as a proxy for racism-related barriers.
